# Human Usutu Virus Infections in Europe: A New Risk on Horizon?

**DOI:** 10.3390/v15010077

**Published:** 2022-12-27

**Authors:** Dániel Cadar, Yannick Simonin

**Affiliations:** 1 Bernhard Nocht Institute for Tropical Medicine, WHO Collaborating Centre for Arbovirus and Hemorrhagic Fever Reference and Research, 20359 Hamburg, Germany; 2 Pathogenesis and Control of Chronic and Emerging Infections, University of Montpellier, INSERM, EFS, 34000 Montpellier, France

**Keywords:** Usutu, flavivirus, neurological disorders

## Abstract

The Usutu virus (USUV), a neurotropic mosquito-borne flavivirus discovered in 1959 in South Africa, has spread over the last twenty years across the European continent. This virus follows an enzootic cycle involving mosquitoes and birds. This caused epizootics with significant bird mortality in Europe in 2016 and 2018. It can also occasionally infect humans and other mammals, including horses and bats, which act as incidental or dead-end hosts. The zoonotic risk associated with this succession of avian epizootics in Europe deserves attention, even if, to date, human cases remain exceptional. Human infection is most often asymptomatic or responsible for mild clinical symptoms. However, human Usutu infections have also been associated with neurological disorders, such as encephalitis and meningoencephalitis. One of the major complexities of the study of USUV pathogenesis is the presence of a great diversity of lineages which could co-circulate spatiotemporally. In this review we discuss several aspects of the circulation of Usutu virus in humans in Europe, the neurological disorders associated, involved viral lineages, and the issues and questions raised by their circulation.

## 1. Structure and Viral Cycle

### 1.1. Structure and Organization

Usutu virus is an emerging encephalitic arbovirus of the *Flaviviridae* family [1]. It belongs to the Japanese encephalitis virus (JEV) serocomplex and it is phylogenetically close to several human and animal pathogens, e.g., West Nile virus (WNV) and Murray Valley encephalitis virus (MVEV) [2,3]. It was first identified in 1959 near the Usutu River in Swaziland, South Africa and was subsequently isolated from field-caught *Culex neavei* mosquitoes by intracerebral inoculation of newborn mice [1,2].

**Figure 1:**
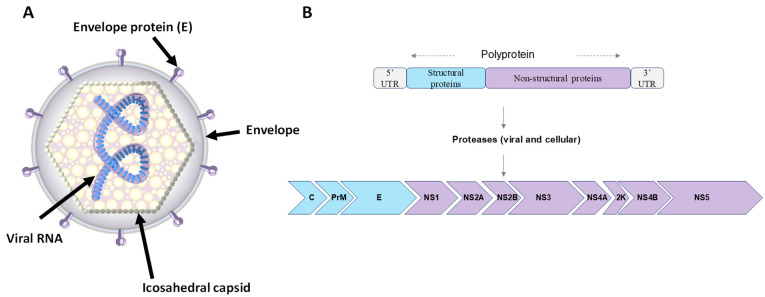
Usutu virus virion structure and genomic organization. (**A**) Schematic representation of USUV virion structure: virion surface is enhanced with the envelope and membrane proteins, anchored in a lipid bilayer with an icosahedral symmetry. (**B**) Representation of the USUV genome. RNA of positive polarity composed of 11 genes. A single reading frame codes for a polyprotein which is cleaved co- and post-translationally by viral and cellular proteases releasing 3 structural proteins (C, prM, and E) and 8 non-structural proteins (NS1, NS2a, NS2b, NS3, NS4a, 2K, NS4b, and NS5).

The USUV particle has a spherical structure with a 40–60 nm diameter and is comprised of an envelope and an icosahedral capsid containing the viral genome (Figure 1). Immature virions have envelope (E) and premembrane (prM) protein heterodimers on their surface, which are cleaved during maturation of the virus particle. Mature virions thus have an envelope composed of structural proteins E and M, which are integrated into a lipid bilayer derived from the endoplasmic reticulum membrane of the host cell, and a capsid composed of protein C [3]. The USUV genome contains a single-stranded RNA molecule of positive polarity coding one long open reading frame (ORF) of approximately 11 kb in length. It is flanked by a type I cap (m7GpppAmN) at 5′, which allows for stabilization and initiation of translation, and lacks a polyadenylated tail (polyA) at 3′ [4]. The genome has a single reading frame encoding a viral polyprotein of about 3400 amino acids, itself flanked by 5′ and 3′ untranslated regions (5′-UTR and 3′-UTR) [5]. This polyprotein is cleaved to release three structural proteins, C, E, and prM, and seven non-structural proteins (Figure 1): NS1 (associates with NS4A to allow genomic replication); NS2A (coordinates the passage of packaged RNA to replication); NS2B (membrane protein, allowing the formation of a protease complex with NS3); NS3 (enzyme with different activities: trypsin-like serine protease, helicase, and RNA triphosphatase, also involved in RNA replication); NS4A (associates with NS1 for genomic replication); NS4B (involved in antiviral resistance); and NS5 (RNA-dependent RNA polymerase (RdRp)) [6].

### 1.2. Viral Cycle

Knowledge of the viral cycle of USUV is mainly based on the previous study of other flaviviruses, such as WNV. The attachment of the virus E protein to a cellular receptor on the plasma membrane is the first step in the viral cycle of flaviviruses [7]. Cell receptors are known to be frequently associated with entry of certain flaviviruses, for example, integrin αvβ3, C-type lectin receptors (mannose receptors or DC-SIGN), or sulfated glycosaminoglycans (heparin sulfate). However, the USUV receptor is not known [7]. A recent study shows that the C-type lectin receptor (CLR), langerin, could be one of the virus receptors in Langerhans cells (LCs) [8]. Endocytosis, mostly clathrin-dependent, allows the entry of virions, then an acidification of the endosomal environment leads to the fusion of the E protein with the endosomal membrane and the release of the capsid into the cytoplasm. This uncoating releases the viral genome and allows for a direct translation of the +RNA into polyprotein by the cellular ribosomes, as if the viral +RNA was cellular mRNA. This polyprotein is then cleaved by cellular proteases and by the NS2B/NS3 complex, leading to the formation of structural and non-structural proteins. Following this translation step, the NS proteins form the replication complex by associating with endoplasmic reticulum membranes and other cellular proteins of the host cell. Replication then takes place, starting with the synthesis of a single-stranded (ss) negative (−) RNA, which serves as a template for the synthesis of ss positive (+) RNAs that transiently form a double-stranded RNA (ds) called dsRNA; this is the replication intermediate [9]. Subsequently, the assembly of genomic RNA and structural proteins at the replicative niche of the endoplasmic reticulum results in the formation of immature virions that can undergo post-translational modifications, such as glycosylation of the viral envelope which plays an important role in the recognition of cellular receptors. Transport into the Golgi apparatus allows for the cleavage of prM to M proteins, by a cellular protease called furin, as well as other post-translational modifications. Finally, the infectious viral particles are released from the cell by exocytosis [9,10,11].

## 2. Transmission Cycle

### 2.1. Transmission Routes

The transmission cycle of USUV involves ornithophilic mosquitoes as vectors, mainly of the genus *Culex*, and birds as amplifying hosts and reservoirs of the virus. It shares many characteristics with WNV [12,13]. Infected mosquitoes will therefore feed preferentially on birds, often species belonging to the genera *Passeriformes* (sparrows, magpies, blackbirds) and *Strigiformes* (owls) [14,15]. Birds infected by the mosquito’s blood meal will develop sufficient viremia to allow transmission of the virus to a new mosquito during a subsequent bite. However, these *Culex* spp. mosquitoes, or gateway vectors such as *Aedes albopictus* mosquitoes, may take their blood meal from a mammalian host, such as a horse, bat, or human, considered as incidental hosts.

### 2.2. Vectors

*Culex pipiens*, an ornithophilic species which can also feed on humans, is the main vector of USUV in Europe. In addition, the vector competence of *Culex pipiens*, *Culex neavei,* and *Culex quinquefasciatus* for USUV have been demonstrated under laboratory conditions [16,17]. Gateway vectors that are rather mammalophilic, such as *Aedes japonicas,* or anthropophilic, such as *Aedes albopictus* [18,19,20], can also be naturally infected, even if their vector competence remains relatively weak. However, these species have been repeatedly found infected in field-collected samples in the Emilia-Romagna region, Northern Italy [16,18,21]. There are currently no data on USUV’s ability to sustain itself in mosquito populations by vertical transmission, unlike WNV.

### 2.3. Reservoirs and Amplifier Hosts

Although considered reservoirs, some avian species are particularly susceptible to USUV infection with a high mortality rate when a naïve population is reached. This is the case for blackbirds (*Turdus merula*), grey owls (*Strix nebulosi*), and sparrows (*Passer domesticus*) [22,23]. The virus and associated necrotic lesions, including neurological lesions, have been identified in many organs of cadavers of these species. Massive mortality of birds through Europe is also indicative of the high pathogenicity of USUV in different avian populations [24,25,26,27]. Several avian species, such as the kestrel (*Falco tinnunculus*) or the babbler (*Sylvia curruca)*, are believed to be responsible for the introduction of USUV into Europe. Subsequently, blackbirds, magpies, or sparrows are believed to have disseminated the virus throughout the continent.

### 2.4. Incidental Hosts

Incidental hosts of USUV and WNV are considered hosts that are not involved in the transmission cycle. They correspond to epidemiological dead ends for viral propagation because the viremia in the infected mammals is not high enough to ensure transmission via mosquito bites. USUV has been identified in dogs, wild boar, and several wild ruminants, such as deer (*Cervus elaphus)*, sheep (*Ovis aries),* and Roe Deer (*Capreolus capreolus)* as well as zoo species [28,29,30,31,32]. Rodents, such as bats, as can also be infected [33,34]. As for WNV, humans, and to a lesser extent horses, are also considered incidental hosts of USUV [32].

## 3. Geographical Distribution

USUV was first discovered in *Culex neavei* mosquitoes in 1959 in South Africa, in Swaziland near the Usutu River [2]. Then, the first human infection was identified in the Central African Republic in 1981, followed by a second in Burkina Faso in 2004 [35]. Subsequently, the virus was identified in several African countries with surveillance programs (Ivory Coast, Morocco, Nigeria, Uganda, and Senegal), suggesting a wider distribution on this continent, especially in countries without adequate surveillance programs [35].

The virus likely reached the European continent for the first time at the end of the 1950s, in Spain, and was reintroduced between 1970 and 1980 (in Italy and Austria) [36]. Retrospective studies suggest that USUV was associated with the deaths of a significant number of birds, including blackbirds in Italy in 1996 [37]. However, it was only in 2001 that the emergence of USUV in Central Europe was confirmed after a significant mortality of blackbirds was reported in Austria [38]. Subsequently, USUV was discovered in an occasional recurrent manner in animals (birds, bats, and horses) and mosquito vectors in several European countries (Belgium, Czech Republic, France, Germany, Hungary, Italy, Spain, and Switzerland), suggesting its endemization in these countries [15,32]. New epizootics were recorded in 2016 and 2018 which affected several European countries, including Belgium, France, Germany, and the Netherlands [15]. USUV was likely dispersed to Europe via migrations of its avian hosts from Africa to Europe, and then further spread via resident wild birds [37]. A second introduction then took place from the Central African Republic to Austria, and more precisely Vienna, where the first avian epizootic was recorded in 2001 [38].

## 4. Classification and Phylogeny

**Figure 2:**
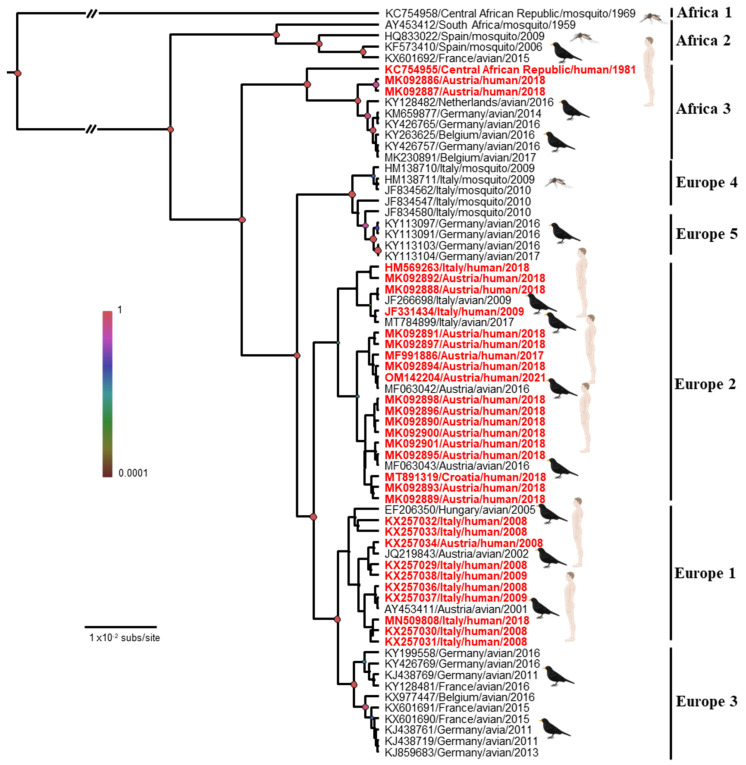
Bayesian maximum clade credibility tree representing the phylogenetic placement of the human Usutu virus cases (in red) compared with some representative USUVs based on partial NS5 gene sequences. Phylogenetic analysis was performed by using Bayesian Markov chain Monte Carlo (MCMC) tree-sampling method implemented in BEAST v.1.8.0 (http://beast.bio.ed.ac.uk, accessed on 20 November 2022). Statistical supports of grouping from Bayesian posterior probabilities (clade credibility) are indicated at the nodes (colored circles). GenBank accession numbers, countries of origin, host, and years of detection of the sequences used to reconstruct the tree are indicated on the branches. Scale bar indicates mean number of nucleotide substitutions per site. Edited with Biorender.com.

The introductions of USUV into Europe follow the main migratory flows: the first introduction from Africa to Spain overlaps with the East Atlantic migratory flow, and that from Africa to Central Europe overlaps with the Black Sea/Mediterranean migratory flow [37]. Therefore, USUV has been classified into eight lineages: African (Africa 1/2/3) or European (Europe 1/2/3/4/5) (Figure 2). Although the majority of strains currently circulating in Europe belong to the European lineages of USUV, African lineages continue to be introduced into the continent, such as the African 2 and 3 lineages discovered in 2018 in *Culex pipiens* in the South of France [39]. The Africa 2 lineage of USUV has also been identified in France in a human patient [40] and in owls in Berlin Zoo in 2015 [41]. The Europe 1 lineage appears to derive from a Senegalese strain that reached Spain and was implicated in the first avian epizootic in Austria in 2001 [38]. The Europe 2 lineage comes from an Austrian strain dating back to 1993. This lineage reappeared during the autochthonous Italian cases of 2009/2010 and the Austrian and Hungarian cases in 2016 [42]. An Italian strain that circulated in 2007 would be the origin of the Europe 3 lineage. This lineage is responsible for the massive bird die-off observed in France in 2015, in Germany in 2011/2013, and in Belgium in 2016 [26,41,43]. The Europe 4 lineage comprises only a few strains, some of which were circulating in Italy in 2010 and 2015 [44]. Finally, the Europe 5 lineage was isolated from infected birds in Germany in 2016 [44]. These different lineages seem to vary in virulence in both humans and birds and deserve further investigation for a better understanding of their distribution and virulence in animals and humans [34,45,46,47].

## 5. Human Infections

### 5.1. Clinical Manifestations

In humans, the incubation period of USUV is estimated at about 3 to 12 days after an infected mosquito bite. This estimation is based on WNV infection as a reference because of the limited clinical data available for USUV infection [48]. After incubation, a short viremic phase is triggered and the patient develops the first symptoms. The virus is then detectable in urine. Seroprevalence studies suggest that USUV is asymptomatic or associated with mild symptoms in most cases. However, the incidence rate of asymptomatic patients is currently unknown. The symptomatic phase of the infection is usually characterized by moderate fever, sometimes associated with rash and febrile jaundice [49]. The zoonotic potential of USUV infection was first described in the African continent. The first two human cases were reported in the Central African Republic in the 1980s and in Burkina Faso in 2004 [35]. Fever and skin rash were reported for both cases. Since, all human cases of USUV infection have been reported in Europe only, although seroprevalence studies conducted in other countries highlight the active circulation of this virus in the African continent [50,51] (Table 1).

The first autochthonous human cases of USUV infection in Europe were reported in 2009 in Italy and manifested as two cases of meningoencephalitis in immunosuppressed patients: one, a woman treated for B-cell lymphoma, and another who had an orthotopic liver transplant [57,73]. Since, most human clinical cases have been identified in Italy likely due to an active circulation of this virus in this country, but also due to an elaborate USUV surveillance program coupled with WNV monitoring. Indeed, in Italy USUV infection is a notifiable disease and USUV surveillance has been included in the national plan since 2017 [74]. Therefore, between 2008 and 2009, 12 additional cases were identified in cerebrospinal fluid (CSF) or blood samples of Italian patients [59,60]. More recently, in 2018, one patient with USUV-associated encephalitis and six with fever were detected during the surveillance period [63] and in 2022 two new cases of febrile USUV infections were detected, while four asymptomatic donors were also identified as positive for the virus [66]. This detection of USUV in blood donors has already been demonstrated before in Italy. Indeed, 38 blood donors were detected as positive in 2017–2018 in several regions of Italy [63,64,65,75]. The consequences for blood donor recipients who receive USUV-positive blood are not yet known. Blood donors in endemic areas who have recently been sick with a clinical illness compatible with an arboviral disease should consider delaying blood donation. Blood donation agencies may want to assess the risk for transmission in endemic areas and consider updating their testing protocols accordingly [61]. Note that there have been reports of blood donors with USUV infections testing positive after screening by the West Nile virus nucleic acid tests (NAT), suggesting potential screening cross-reactivity between these two related viruses [61].

Cases of human USUV infection have also been detected in other European countries, although the surveillance networks in place are often not as efficient. Three neurological cases were reported in Croatia in 2013: one patient presented with meningitis and two with meningoencephalitis. [76]. In 2018, three other neurological diseases were identified in Croatia, as well as a case in the Czech Republic and another one in Hungary [55,56,67]. In 2020, one case was identified in Switzerland by metagenomic next-generation sequencing [70]. Two large studies conducted in Austria on blood donors have also identified 24 positive donors [52,72] and a patient with meningitis [53]. In France, USUV infection was reported in 2016 in a patient with idiopathic facial paralysis, and one febrile case was detected in 2022 [40,54].

Seroprevalence studies indicate a non-negligible exposure of humans to USUV infection risk (Table 2). These studies have been carried out in Austria, Germany, Italy, France, Hungary, the Netherlands, Romania, and Serbia, reporting an USUV antibody prevalence between 0.02% and 3% among healthy blood donors [32,62,69,71,72,75,77,78,79,80,81,82]. Higher prevalence (6/7%) was found in more exposed populations, such as forestry workers [60,78,83]. Several of these studies show that USUV circulates more actively in humans than WNV in Europe, and prevalence studies of USUV infection in mosquito vectors in Europe also highlight a higher level of exposure to USUV than to WNV [18,84,85]. However, seroprevalence data are still very scarce for assessing the real incidence of USUV in humans, and to date, there are no efficient serological diagnostic tools for large-scale screening. The technical disadvantages of serology testing are indeed confronted. This is often the case for flaviviruses due to lack of specificity linked to the substantial antigenic cross-reactions with representative flaviviruses, meaning results often require confirmation by the more fastidious seroneutralization approach [13].

**Figure 3:**
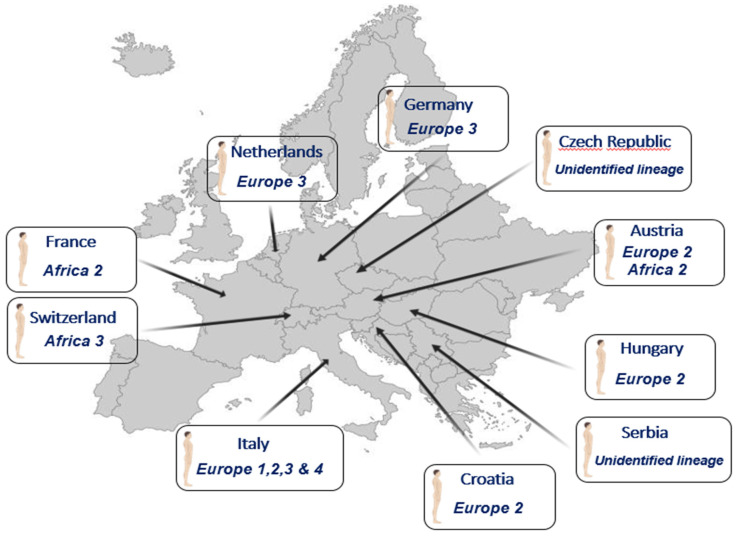
Human cases of Usutu virus infection and the lineages involved.

Although the USUV lineages involved in most of the studies remain unknown, few studies showed that several lineages circulate in the human population (Figure 3).

We can nevertheless note that infections due to the Europe 2 lineage are over-represented in the human cases with neurological diseases (Table 1). Since the appearance of the EU2 lineage in 2014 in Germany, initially detected in a blackbird [86], this lineage has become the dominant circulating USUV in several countries, such as Austria and Hungary [87]. In addition, several studies have reported increased virulence of this lineage. In particular, a study using chicken embryos showed the presence of viral antigens in the brains of infected embryos. These chicken embryos subsequently died following infection with a USUV EU2 isolate [47]. The EU2 lineage has also shown elevated virulence in mouse models and human neuronal cells [45]. Point mutation at position 835 in NS5 has been found in several EU2 strains [45]. This mutation was also found in the first reported human case in Europe from 2009 (associated with neurological complication), and it has also been associated with neuroinvasive capacity in JEV, WNV, Kunjin virus (KUNV), and Murray Valley encephalitis virus (MVEV) [57,88]. Furthermore, mutation at position 835 in the viral polymerase is located in the RdRp domain, thus most likely increasing the viral fitness by replication efficiency, which could result, in vivo, in an exacerbated virulence phenotype [15]. Finally, a point amino acid substitution of glutamic acid for aspartic acid (E460D) in NS5 has demonstrated a role in the virulence of tick-borne encephalitis virus (TBEV) in mice [89]. Further studies are needed to evaluate if the over-representation of the EU2 lineage in acute infections and in neurological diseases is related to a higher virulence of this lineage or other reasons (vector competence, multiplication in the reservoir host, etc.).

Overall, to date, over 100 cases of acute human infection have been described in Europe, including 30 patients with neurological symptoms. Concerning the neurological disorders for which we have detailed information, we can notice that most patients have had comorbidities of varying severity, but that there are also patients developing neurological forms without identified concomitant pathological conditions (Table 3). Nevertheless, the number of cases reported to date is not sufficient for determining age or gender criteria in the exposure to neurological risks associated with USUV. Furthermore, it is still unknown whether these human cases represent the tip of the iceberg or the incidence of acute USUV infection with mild symptomatology remains minimal since the diagnosis of USUV infection is rarely considered. The emergence of avian epizootics is synchronous with a greater exposure of humans to zoonotic risks. In this light, the human cases detected in Italy were concomitant with an epizootic outbreak of USUV showing that humans are exposed to a higher zoonotic pressure than in that of WNV [79]. Similarly, the human cases detected from 2016 to 2018 were simultaneous with the most epizootics observed in Europe [15,58,72]. Phylogenetic analysis of the strains detected in humans in Germany and Austria confirmed that they were strains of the Europe 2 and 3 lineages, that had indeed circulated among blackbird and passerine populations at that time. Similarly, a different strain from the Africa 2 lineage that was detected in a human case in the south of France in Montpellier [40] was also simultaneously identified in pools of mosquitoes captured nearby, in the Camargue area [39]. Hence, significant circulation of USUV in both reservoirs and vectors, at a given time and place, increases the probability of human cases.

### 5.2. Diagnosis

Diagnosis of USUV infection is classically made by detection of specific antibodies, by demonstration of the viral genome, genomic fragments, or by cell culture. Direct diagnosis is based on the detection of viral RNA in blood and CSF by reverse polymerase chain reaction (RT-PCR). Indirect or incidental detection of the virus occurs in most of the blood donor cases which cross-react with WNV in the COBAS systems used in several national surveillance programs for WNV screening. It can also be performed in some cases by cell culture. Many cell types tolerate viral replication. The cells mainly used are C636 insect cells or Vero cells, in which the virus develops a cytopathic effect, but many alternatives are possible [90]. However, this direct detection method is not commonly used, as infection with these viruses usually results in a short-lived viremia as humans are an epidemiological dead-end for these viruses. Instead, serological tests are performed by ELISA or approaches using immunofluorescence. These tests suffer from a lack of specificity and must be systematically controlled by neutralization approaches to exclude systematic cross-reactions obtained with antibodies directed against other representatives of flaviviruses, particularly between WNV and USUV, which are phylogenetically closely related. To date, no commercial diagnostic test is available to detect USUV, unlike WNV.

### 5.3. Treatment and Prevention

There is no vaccine against USUV, although an experimental DNA vaccine appeared to be protective in mice [68]. Given that USUV and WNV co-circulate in the same geographic regions, the question arises of cross-protection or an antibody-dependent enhancement (ADE) effect for USUV and WNV (or other flaviviruses). Studies in mice suggest partial post-flavivirus cross-protection in mice [91].

To limit USUV infection, as for infection with other arboviruses, there is no specific treatment. Favipiravir, a viral RNA polymerase inhibitor, has been shown effective in mice [92]. Supportive and symptomatic treatments may be used, such as paracetamol for pain and/or fever, hydration (orally or sometimes by injection), and antiemetics. Patients who develop meningoencephalitis or encephalitis may need to be monitored for elevated intracranial pressure and/or seizures. Although many efforts are being made to find specific or global treatments against arboviruses, the most widely used prevention methods are still based on vector control. Vector control is mainly based on the use of repellents and mosquito nets, or the use of insecticides which nevertheless have limitations, such as the resistance developed by arthropods against insecticides. To prevent mosquito breeding, stagnant water should be periodically removed from containers around homes.

## 6. Conclusions

The study of the circulation of USUV is not well documented. There are still many unknown aspects, especially concerning reservoirs and the real prevalence in humans. A better understanding of the circulation and risks associated with these viruses, and more globally with arboviruses, requires a review of our monitoring strategy, through the implementation of the One Health global approach. This approach consists of studying the interactions between animals, humans, and their various environmental factors (e.g., climatic condition, vector population dynamics) by setting up a transdisciplinary and multidisciplinary approach involving many actors. These include, for instance, human health professionals (e.g., physicians, pharmacists, virologists, epidemiologists), but also with the participation of professionals from the veterinary field and entomologists. The objective is to implement efficient surveillance programs and preparedness strategies for emerging diseases like USUV.

## Figures and Tables

**Table 1:** Description of human Usutu cases worldwide and associated viral lineages. In red: human USUV cases associated with neurological disorders.

Acute Infections (n = 112) 30 Neuroinvasive Diseases							
Country	Year	Cases	Sample Type	Symptomatology	Studied Population	USUV Lineages	Ref
**Burkina Faso**	2004	1	Blood	Fever and jaundice	Clinical case	ND	[35]
**CAR**	1981	1	Blood	Eruptive fever	Clinical case	AF3	[35]
**Austria**	2017	6/12047	Blood	Healthy	Blood donors	EU2	[52]
	2018	18/31598	Blood	Healthy	Blood donors	EU2/AF3	[53]
	2021	1	CSF	Meningitis	Clinical case	EU2	[54]
**Croatia**	2013	3/95	Blood	Meningoencephalitis	Meningoencephalitis patients	ND	[55]
	2018	3/178	Blood /Urine	Neuroinvasive disease included 1 fatal meningoencephalitis	Neuroinvasive cohort	EU2	[56]
**Italy**	2009	1	CSF	Meningoencephalitis	Clinical case	EU1	[57]
	2009	1	Blood	Encephalitis	Clinical case	EU2	[58]
	2008–2009	3/44	CSF	Meningoencephalitis	Meningoencephalitis patients	ND	[59]
	2008–2011	8/306 + 2/609	CSF/blood	Meningoencephalitis /healthy	Meningoencephalitis patients + various healthy and sick subjects	ND	[60]
	2016–2018	25/73964	Blood	Healthy	Blood donors	ND	[61]
	2017–2018	9	Blood	Healthy	Blood donors	EU2/EU3/EU4	[62]
	2017–2018	8	Blood/Urine	1 encephalitis, 6with fever and 1 viremic blood donor	Suspected autochthonous arboviral infection and positive blood donors	EU2	[63]
	2018	2/8889	Blood	Healthy	Blood donors	EU2	[64]
	2018	1/44	Blood	Healthy	Blood donors	EU1	[65]
	2022	6	Blood	4 healthy, 2 fever	Clinical case	ND	[66]
**Czech Republic**	2018	1	Blood	Meningoencephalitis	Clinical case	ND	[67]
**Germany**	2016	1	Blood	Healthy	Blood donors	EU3	[68]
**France**	2016	1/666	CSF	Idiopathic facial paralysis	Patients with infectious and/or neurological signs	AF2	[40]
	2022	1	Blood	Fever	Clinical case	ND	[69]
**Hungary**	2018	1	Blood	Aseptic meningitis	Clinical case	EU2	[70]
**Netherlands**	2018	7/12,040	Blood	Healthy	Blood donors	EU3	[71]
**Switzerland**	2020	1/25	Blood	Disseminated fungal infection (fatal case)	Clinical case	AF3	[72]

CAR: Central African Republic.

**Table 2:** Seroprevalence studies of USUV in the European populations.

n = 127					
Country	Year	Number	Prevalence	Studied Population	Ref
**France**	2019–2020	15/500	3%	Outpatients	[32]
**Germany**	2012	1/4200	0.02%	Blood donors	[80]
**Hungary**	2019	5/3005	0.17%	Blood donors	[81]
**Italy**	2009	4/359	1.1%	Blood donors	[77]
	2008–2011	40/609	6.5%	Healthy/various sick subjects	[60]
	2010–2011	14/6000	0.23%	Blood donors	[78]
	2012	24/3069	0.78%	Blood donors	[79]
	2014–2015	6/33	18.1%	Healthy forestry worker	[82]
	2014–2015	2/200	1%	Blood donors	[78]
	2018	2/8889	0.02%	Blood donors	[64]
**Netherlands**	2018	7/12,040	0.06%	Blood donors	[71]
**Romania**	2018	0/1200	0.00%	Blood donors	[82]
**Serbia**	2015	7/93	7.5%	Healthy exposed subjects	[83]

**Table 3:** Description of clinical case with identified neurological damage. NC: Not Communicated.

Country	Clinic	Age (Years)	Gender	Health Condition/Comorbidity	Outcome
**Austria**	Meningitis	81	Man	Hypertension, left-sided hemifacial spasms	Constant monitoring and hospital care (7 months after hospitalisation)
**Croatia**	Meningoencephalitis	29	Woman	Healthy	NC
		61	Man	Healthy	NC
		56	Man	Arterial hypertension, coronary heart disease, hyperlipidemia, and diabetes	NC
	Neuroinvasive disease	25	NC	Healthy	NC
		84	NC	Healthy	NC
		60	NC	Immunocompromised. Chronic lymphocytic leukemia	Fatal
**Czech** **Republic**	Meningoencephalitis	46	Woman	Healthy	Patient discharged after 12th day of hospitalisation.
**France**	Idiopathic facial paralysis	39	Man	Healthy	Patient discharged 3 days after hospitalisation. Symptoms disappeared within a few weeks.
**Hungary**	Meningitis	40s	Man	Healthy	Duration of hospitalisation was 7 days
**Italy**	Meningoencephalitis	60s	Woman	Immunocompromised. Large B cell lymphoma.	NC
	Encephalitis	40s	Woman	Immunocompromised. Orthotropic liver transplantantation	Fulminant hepatitis, coma. The patient slowly regained motor function
	Meningoencephalitis	40	Man	Chronic liver disease	NC
		73	Man	Chronic obstructive pulmonary disease, diabetes	NC
	Encephalitis	54	Woman	Polyneuritis, hypertension	NC
	Encephalitis	60s	Man	Hypertension, diabetis	NC
	Encephalitis	67	Woman	Aortic and mitral insufficiency	NC
